# Fibronectin Functionalized Electrospun Fibers by Using Benign Solvents: Best Way to Achieve Effective Functionalization

**DOI:** 10.3389/fbioe.2019.00068

**Published:** 2019-04-03

**Authors:** Liliana Liverani, Manuela S. Killian, Aldo R. Boccaccini

**Affiliations:** ^1^Institute of Biomaterials, Department of Materials Science and Engineering, University of Erlangen-Nuremberg, Erlangen, Germany; ^2^Chair for Surface Science and Corrosion, Department of Materials Science and Engineering, University of Erlangen-Nuremberg, Erlangen, Germany

**Keywords:** electrospinning, benign solvents, nanofibers, fibronectin, functionalization, scaffolds, tissue engineering

## Abstract

The aim of this study is to demonstrate the feasibility of different functionalization methods for electrospun fibers developed using benign solvents. In particular three different approaches were investigated to achieve the functionalization of poly(epsilon caprolactone) (PCL) electrospun fibers with fibronectin. Protein surface entrapment, chemical functionalization and coaxial electrospinning were performed and compared. Moreover, bilayered scaffolds, with a top patterned and functionalized layer with fibronectin and a randomly oriented not functionalized layer were fabricated, demonstrating the versatility of the use of benign solvents for electrospinning also for the fabrication of complex graded structures. Besides the characterization of the morphology of the obtained scaffolds, ATR-FTIR and ToF-SIMS were used for the surface characterization of the functionalized fibers. Cell adhesion and proliferation were also investigated by using ST-2 cells. Positive results were obtained from all functionalized scaffolds and the most promising results were obtained with bilayered scaffolds, in terms of cells infiltration inside the fibrous structure.

## Introduction

The electrospinning technique is a well-developed method for the fabrication of nano- and microfibers suitable for several applications in the biomedical field because such fibrous structures mimic the morphology of the extra cellular matrix (Braghirolli et al., 2014; Kishan and Cosgriff-Hernandez, 2017; Kitsara et al., 2017). Recently, electrospinning involving the use of benign solvents, respect than the standard solvents used for this technique (i.e., chloroform, dichloromethane, hexafluoroisopropanol, etc.) has become a topic which has arisen the interest of the scientific community (Sun et al., 2010; Krishnan et al., 2013; Liverani et al., 2017b).

The use of the terms “benign solvents” is related to several topics and their applications are not restricted only to the electrospinning process. In fact, even though an official definition was not reported till now, benign solvents are mentioned for the chemical product design (Hada et al., 2013), roll-to-roll processing for flexible organic electronics (Roth et al., 2015) and synthesis of organic carbonates (Heyn, 2015). For most of those applications, the words “benign” and “green” are used as synonyms to identify solvents with the following characteristics: high boiling point, low vapor pressure, non-toxic, able to dissolve a great range of organic compounds, inexpensive and recyclable. An exception of the use of “benign” and “green” as synonyms is represented by the field of electrospinning, in which the definition of green electrospinning implies the use of emulsions and not solvents.

Therefore, benign solvents for the electrospinning can be clearly defined as solvents with reduced or absent toxicity for humans [Class 3 according to ICH guidelines (ICH, 2018)], suitable for the electrospinning process implying the ability to solve polymers and blends, maintaining their electrospinnability, avoiding protein denaturation, and the presence of their traces in electrospun mats is not affecting the sample biocompatibility. Benign solvents are also environmentally friendly, enhancing aspects related to lab worker safety and waste management.

In particular, the use of benign solvents does not limit the applications of electrospun fibers in comparison with the same fibers obtained by using standard solvents. The use of benign solvents has been already reported for the fabrication of blended and composite fibers for tissue engineering applications (Gönen et al., 2016; Lepry et al., 2016; Liverani et al., 2017a). Their use resulted beneficial in particular for the fabrication of fibers starting from proteins which could easily degrade in standard solvents (Zeugolis et al., 2008; Chakrapani et al., 2012).

Fibronectin is one of the extracellular matrix proteins, in particular it is a ligand-integrin affinity protein, widely used for the functionalization of electrospun scaffolds (Mukhatyar et al., 2011; Campos et al., 2014; Chutipakdeevong et al., 2014; Regis et al., 2014; Zuidema et al., 2014), but also for the coating of cell culture plates to promote cell adhesion and spreading, in already commercially available products (Hu et al., 2018; Sherman and Rothenberg, 2018).

In literature, electrospun mats have been functionalized with fibronectin, but also with other relevant proteins as laminin and cadherin. The most common ways to obtain functionalization could be grouped in two categories: one related to post treatment of the samples, in this case the functionalization process is not related to the electrospinning process [like protein surface entrapment (Campos et al., 2014) and protein chemical bonding (Chutipakdeevong et al., 2014; Regis et al., 2014; Tallawi et al., 2015; Tortora et al., 2016)] and the other is to achieve fiber functionalization as part of the electrospinning process, like coaxial electrospinning.

Besides scaffold chemical functionalization another important issue for the success of tissue engineering approach is the achievement of an effective cell colonization of the scaffold. In fact, due to the density of electrospun fibers nets, cell infiltration is often limited. To overcome this limitation, several techniques have been investigated (Rnjak-Kovacina and Weiss, 2011) and the use of patterned electrospun structures with macropores is promising. The use of these constructs for the fabrication of more complex multilayered scaffolds with gradients in morphology and chemical signals represents an interesting approach also for the fabrication of scaffolds for interface tissue engineering applications (Seidi et al., 2011).

The aim of the present work is to demonstrate the suitability of electrospun mats obtained with benign solvents to be processed for standard functionalization techniques. A comparative analysis in terms of protein adsorption and cells response was performed among the different functionalization methods investigated. As proof of concept of the functionalization methods versatility and suitability for electrospun macro patterned samples, they were applied on bilayered scaffolds obtained by overlapping two electrospun mats, one with a macro patterned structure, obtained with a similar protocol developed in a previous work (Liverani and Boccaccini, 2016) while the other layer was made of randomly oriented PCL electrospun fibers. The differences in protein coatings were assessed with ToF-SIMS. The spectra of coated fibers were evaluated using principle component analysis (PCA). This method has been widely used for the interpretation of ToF-SIMS data, including analysis of protein samples (Wagner and Castner, 2001). PCA can help to identify the major sources of differences within a sample or between samples, determine where certain compounds exist on a sample, or verify the presence of compounds that have been engineered onto the surface (Graham and Castner, 2012).

## Materials and Methods

### Electrospinning Process

The electrospinning of PCL fibers was performed according to a previous work (Liverani and Boccaccini, 2016) by using a device with a climate control chamber EC-CLI (IME Medical Electrospinning). Briefly, a solution of 20% w/v of PCL (80 kDa, Sigma Aldrich) in glacial acetic acid (VWR) was stirred overnight and then put in an ultrasound bath for 1 h prior to electrospinning. The solution was fed through a 23G needle with a flow rate of 0.4 mL/h, the applied voltage was +13 kV at the needle and −2 kV at the rotating drum (500 rpm rotating speed), the distance between needle and collector was set at 11 cm. Moreover, a gas shield module was used to optimize the Taylor cone, using distilled water as solvent and nitrogen flow of 8 mL/min. The environmental conditions of the electrospinning chamber were set at 23°C and 40% relative humidity. The obtained sample is labeled “PCL AA.” The patterned samples were obtained by using a patterned collector according to a previously used device (Liverani and Boccaccini, 2016). In order to compare the electrospun fibers obtained by using benign solvents with the one produced with standard solvents, a solution of 10%w/v of PCL in a mixture of dichloromethane (DCM) and methanol (MeOH) (in ratio 7:3) was electrospun with the same setup. Solution and process parameters were adapted from Doergens et al. (2015). Briefly, the solution was extruded with a flow rate of 1.5 mL/h, with a needle of 23G, the tip-target distance was set at 11 cm and the applied voltage was +13 kV at the needle and −2 kV at the rotating drum (500 rpm rotating speed). Gas shield setting and environmental parameters were fixed at the same values of the other samples. The obtained electrospun mats is labeled “PCL DCM/MeOH.”

### Functionalization Protocols

Fibronectin (from bovine plasma, solution 0.05 M in tris-buffered saline (TBS), product code F1141, Sigma Aldrich) was diluted with Phosphate Buffer Solution (PBS) to achieve the concentration of 10 μg/mL. This concentration was selected, according to previous works related to the functionalization of electrospun fibers with fibronectin (Campos et al., 2014; Regis et al., 2014). Three different protocols were used to compare different functionalization methods.

The first method was adapted from a literature protocol (Campos et al., 2014). Briefly, PCL electrospun mats were immersed in NaOH (VWR) aqueous solution 0.01 M for 20 min at 37°C. The hydrolyzed mats (labeled as PCL Hy) were rinsed with PBS two times for 5 min each time and then immersed in a fibronectin solution 10 μg/mL (mentioned above) for 24 h at 37°C. The samples (labeled as PCL Hy_FN) were washed with PBS and kept in PBS until further characterization. The samples were not stored in these conditions and prepared fresh just before their characterization to prevent the drying of the functionalized samples surface, which could induce protein denaturation (Killian et al., 2018).

The second method adapted from the protocol of Chutipakdeevong et al. (2014) and Tortora et al. (2016) started with the hydrolysis, using the same conditions explained for the first method. Then the samples were immersed in HCl (VWR) aqueous solution 0.01 M for 30 min at room temperature. The surface activation was achieved by immersing the PCL mats for 1 h in a solution containing 75 mg/mL of N-(3-Dimethylaminopropyl)-N′-ethylcarbodiimide hydrochloride (EDC) (Merck) and 1.15 mg/mL of N-Hydroxysuccinimide (NHS) in 0.1 M 2-(N-Morpholino)ethanesulfonic acid hemisodium salt (MES) buffer. After the rinsing in MES buffer, the functionalization and further steps were performed according to the protocol used for the first method and mentioned above, in order to compare the functionalization techniques. The samples were labeled as PCL EN after the rinsing in MES buffer and PCL EN_FN after the functionalization with fibronectin.

The third investigated method involved coaxial electrospinning. On this purpose, the coaxial needle (EM-CAX, IME Medical Electrospinning) was used in the same setup mentioned above. In particular, the same solution of PCL in acetic acid (previously described) was used for the core and fibronectin solution (10 μg/mL in PBS) was fed in the shell. The process parameters were kept constant with respect to the above mentioned process for the fabrication of PCL mats and the flow rate for the fibronectin solution was set at 10 μL/min. The obtained samples were labeled as “coaxial.”

For the bilayered scaffolds, patterned PCL electrospun mats were functionalized with both the methods previously described and then overlapped with randomly oriented PCL mats untreated (neither hydrolyzed, nor functionalized) to obtain a graded functionalized scaffold. The scaffolds with the upper patterned layer functionalized with the first method were labeled as “Bilayer Hy_FN,” while the bilayered samples with the upper layer functionalized with the second method were labeled as “Bilayer EN_FN.”

### Fibers Characterization

#### Morphological and Chemical Characterization

Morphological evaluation of the electrospun mats was performed by using SEM analysis (Auriga 0750, Zeiss). Before the analysis, the samples were gold coated with a Sputter Coater (Q150T, Quorum Technologies). Calculation of fiber diameters and porosity was obtained with the software ImageJ and its tools (Schneider et al., 2012). ATR-FTIR analysis was used to assess the sample functionalization, comparing the different methods, by using IRAffinity-1S (Shimadzu) spectrophotometer with a resolution of 4 cm^−1^ and 40 scans in wavenumber range of 4,000–400 cm^−1^. The mechanical properties of the electrospun samples obtained with the solvent system DCM/MeOH (PCL DCM/MeOH sample) were assessed by using an universal testing machine Instron 5967, equipped with 100 N load cell at a crosshead speed of 10 mm/min. Samples were cut in strips of 0.5 × 4 cm^2^ and fixed on a paper framework, with internal length of 2 cm, as described in a previous work (Liverani and Boccaccini, 2016).

#### Surface Analysis

ToF-SIMS analysis was performed to characterize all functionalized samples to confirm the presence of fibronectin and the efficacy of the functionalization. In case of the bilayered scaffolds, ToF-SIMS was used to map the protein, confirming the gradient between the top and bottom layer. Positive and negative static SIMS measurements were performed on a ToF.SIMS 5 spectrometer (ION-TOF, Münster). The samples were irradiated with a pulsed 25 keV $Bi_{3}^{+}$ liquid-metal ion beam. Spectra were recorded in the high mass resolution mode (m / Δm > 8,000 at ^29^Si). The beam was electrodynamically bunched down to <1 ns in order to increase the mass resolution and rastered over a 100 × 100 μm^2^ area with five spots per sample. The primary ion dose density (PIDD) was kept at 5 × 10^11^ ions^*^cm^−2^, ensuring static conditions. Images were recorded on three spots (250 × 250 μm) per sample in high lateral resolution mode. Low energy electron flooding was used to prevent charging of the samples. The peak lists used for evaluation were derived from Killian et al. (2018) and are shown in Supplementary Table 1. Signals were identified using the accurate mass as well as their isotopic pattern. Poisson correction was used for integration of the signal intensities. Spectra were normalized to their total intensity and calibrated to ${\text{CH}}_{\text{3}}^{+}$, ${{\text{C}}_{\text{2}}\text{H}}_{\text{3}}^{+}$, ${{\text{C}}_{\text{3}}\text{H}}_{\text{5}}^{+}$, ${{\text{C}}_{\text{4}}\text{H}}_{\text{9}}^{+}$, and ${{\text{C}}_{\text{7}}\text{H}}_{7}^{+}$ signals in positive and ${\text{CH}}_{\text{2}}^{-}$, ${\text{C}}_{\text{2}}^{-}$, CN^−^, and CNO^−^ in negative polarity. Principle component analysis of the spectra was conducted with the software “spectragui” of the NESAC/BIO toolbox (NESAC/BIO, 2014). Peaks were normalized to the total intensity of the PCA peak set to eliminate any systematic differences in total secondary ion yield (absolute intensity) between spectra. The data set was also square root mean-centered to ensure the differences in samples were due to variations around the means and not the variance of the means (Vandeginste et al., 1998).

#### Release of Fibronectin

PCL EN_FN, PCL Hy_FN, and coaxial samples were fixed on appropriate holders (CellCrownTM 24 inserts, Sigma Aldrich) and immersed in PBS. After 30 min, 1, 2, 4, 6, 24, and 168 h (7 days) 1 mL of extract solution was removed and 1 mL of fresh PBS was replenished. For each time point the measurements were performed in triplicate. The amount of fibronectin was evaluated by using Micro BCA Protein Assay Kit (Thermo Scientific) for the colorimetric detection and quantitation of protein with a linear working range of 0.5–20 μg/mL. The calibration was performed on stock solutions of fibronectin in the range 2.5–20 μg/mL. After following the protocol for the sample preparation given by the provider, the samples were analyzed with a UV-vis spectrophotometer (Specord 40, Analytik Jena), by reading at 560 nm. Additionally, fibers morphology was investigated by SEM after 1 and 7 days of immersion in PBS to investigate possible morphological modifications related to the protein release.

#### Biological Assay

Cell studies to assess cell viability, proliferation and morphology were performed by using bone murine stromal cells ST-2 cell line (Leibniz-Institut DSMZ – German Collection of Microorganisns and Cell Cultures GmbH, Germany). All the samples were fixed on a holder for 24 multiwell (Scaffdex, Sigma) and UV exposure for 1 h was used as disinfection method. Before the seeding, ST-2 cells were cultured in RPMI 1640 medium (Thermo Fisher Scientific), supplemented with 10% fetal bovine serum (Lonza) and 1% penicillin/streptomycin (Lonza) and incubated at 37°C with 5% CO_2_. Drop seeding was performed by using an inoculum ratio of 1.5·10^5^ cells/mL with a drop of 100 μl per sample. After 15 min of incubation, 1 mL of RPMI medium was added to each well. WST-8 assay ((2-(2-methoxy-4-nitrophenyl)-3-(4-nitrophenyl)-5-(2,4-disulfophenyl)-2H-tetrazolium, monosodium salt), Sigma), which allows sensitive colorimetric assay for the determination of cell viability in cell proliferation and cytotoxicity assay, was performed 1 day and 7 days after the seeding in triplicate on all the samples, according to the protocol (Liverani et al., 2017a). ANOVA one-way analysis was performed to evaluate the results of cell viability. A *p*-value < 0.05 was considered statistically significant.

Cell morphology was assessed 7 days after the seeding, by staining with rhodamine phalloidin and DAPI (ThermoFisher Scientific). Briefly, a fixation solution with 1,4-piperazinediethanesulfonic acid buffer, ethylene glycol tetraacetic acid, polyethylene glycol, paraformaldehyde, PBS, and sodium hydroxide (Sigma) was used for sample fixation, then the samples were rinsed with PBS and immersed in a permeabilization buffer containing Triton X-100, sucrose and PBS (Sigma). Rhodamine phalloidin solution (8 μL/mL) and DAPI solution (1 μL/mL) were used for the staining. All the samples were then characterized with a fluorescence microscope (Axio Scope A1, Zeiss).

## Results and Discussion

### Morphological Analysis

**Figure 1:**
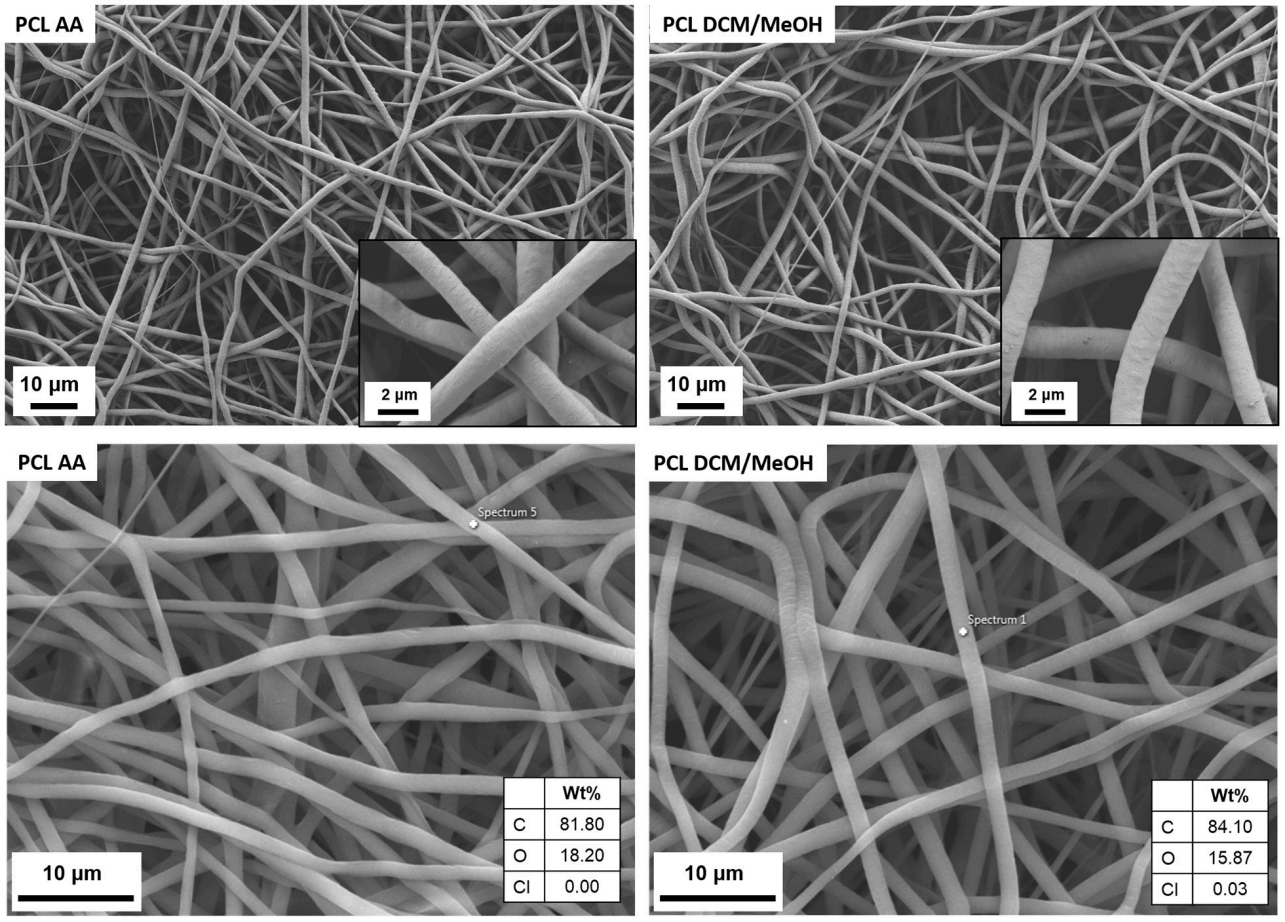
SEM micrographs at different magnifications of PCL electrospun fibers obtained by using acetic acid (PCL AA) and dichloromethane/methanol (PCL DCM/MeOH) as solvents. EDX analysis is reported at the bottom for both samples.

An initial assessment on the effects on fiber morphology related to the electrospinning solvent system was performed. In fact, fibers obtained from benign solvent (i.e., acetic acid) and from a mixture of dichloromethane and methanol were compared and their morphology is shown in Figure 1.

It is possible to notice that for both solvent systems homogeneous bead-free fibers were obtained and with the micrographs at higher magnification is also possible to check that the fiber surface presents the same morphology for both solvents. Moreover, the average fiber diameter was also comparable and the measured values were 1.3 ± 0.2 and 1.6 ± 0.2 μm, respectively, for PCL AA and PCL DCM/MeOH. Additionally, in the bottom part of Figure 1 the EDX analysis on the obtained fibers shows the presence of traces of Cl just on the surface of PCL DCM/MeOH fiber, confirming that residual of solvent could be detected on the sample. The use of benign solvents, as acetic acid does not led to the detection of solvent traces on the fibers. For what concerns the mechanical properties, PCL DCM/MeOH samples had an average Young's modulus of 3 ± 1 MPa, showing values comparable with PCL fibers obtained with standard solvents, as reported in literature (Croisier et al., 2012), but showing lower values respect to the PCL fibers obtained with benign solvents (Liverani and Boccaccini, 2016). This difference could be due to the sub-optimized process parameters adapted from the literature (Doergens et al., 2015) which led to the fabrication of a thin layer of fibers (average sample thickness of 12 ± 4 μm), keeping constant the deposition time respect to the PCL AA.

**Figure 2:**
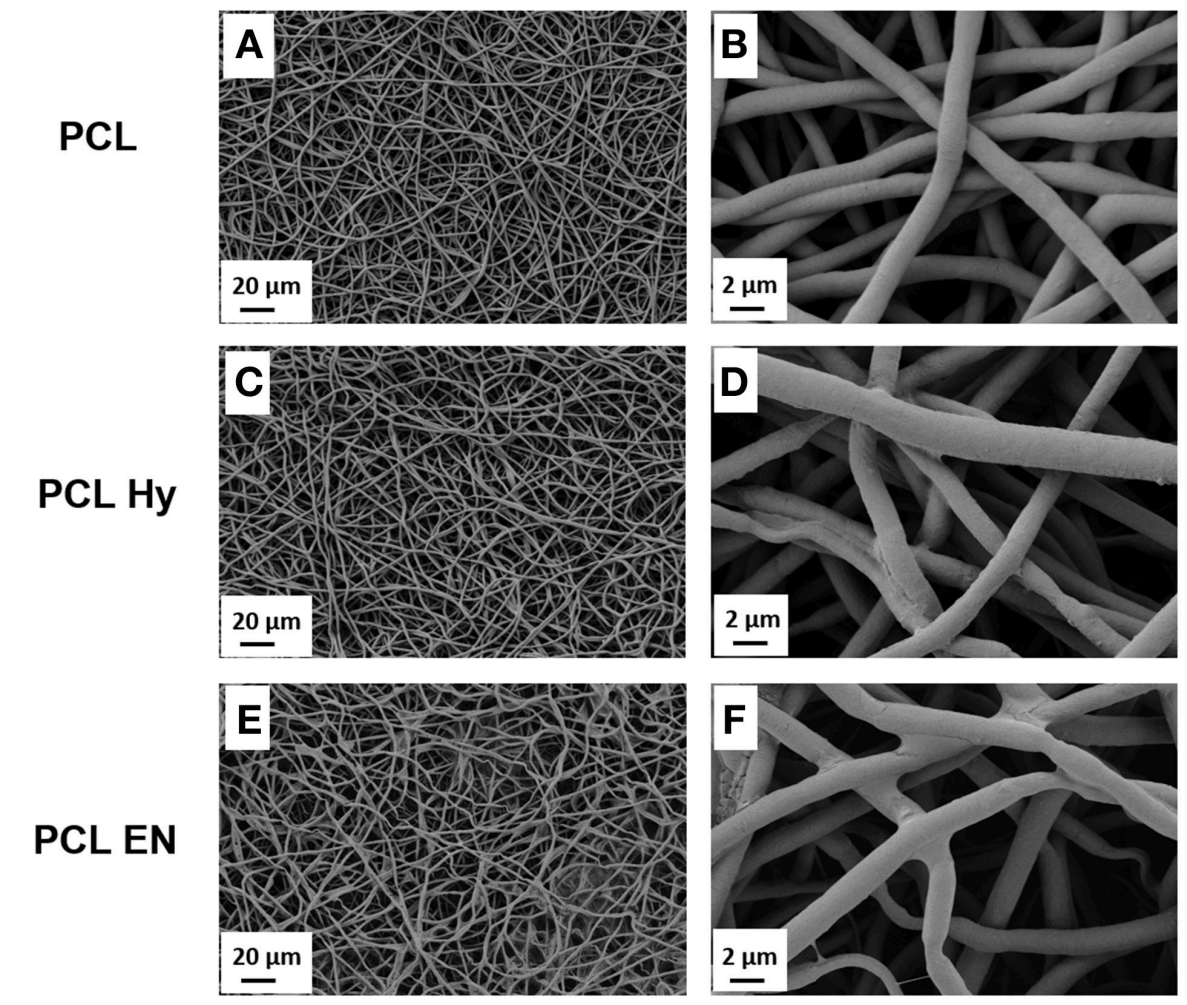
SEM micrographs of PCL fibers before **(A,B)**, after hydrolysis and protonation **(C,D)**, and after EDC-NHS treatment **(E,F)**.

Electrospun fibers morphology was assessed before and after the hydrolysis, protonation and EDC-NHS treatment. The morphology of untreated neat PCL electrospun mats labeled as “PCL” is shown in Figures 2A,B, while the PCL mats after hydrolysis labeled as “PCL Hy” are reported in Figures 2C,D and the PCL mats after EDC-NHS treatment, label as “PCL EN” are reported in Figures 2E,F. For all samples, the typical fibrillary morphology of “PCL” is preserved and the average fiber diameter was not affected by those treatments.

**Figure 3:**
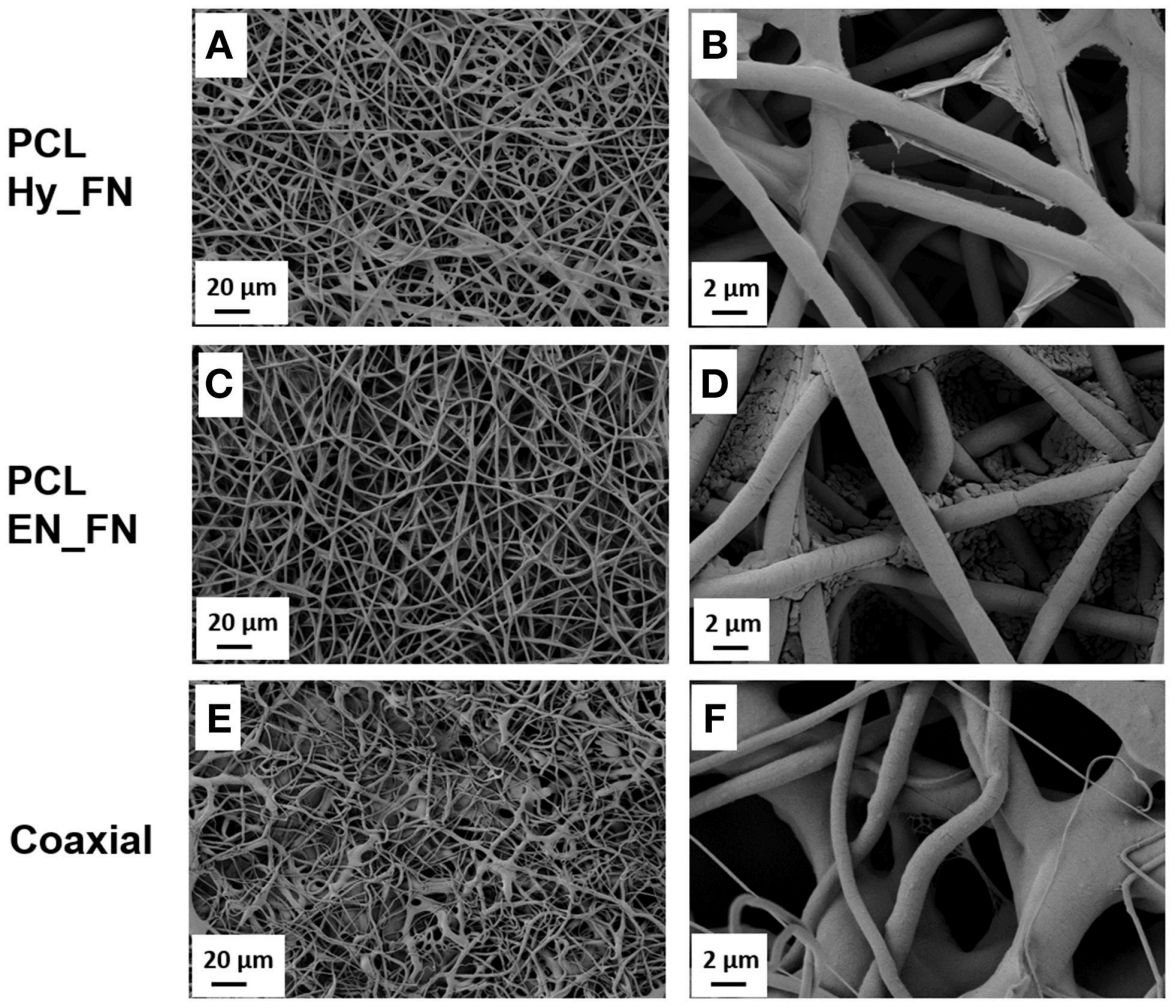
SEM micrographs of PCL fibers before **(A,B)**, after hydrolysis and protonation **(C,D)**, and after EDC-NHS treatment **(E,F)**.

After the functionalization with fibronectin the fibers morphology was assessed and compared. The coaxial fibers were included in this comparison, as shown in Figure 3. In Figure 3A and in more details in Figure 3B, it is possible to notice for the sample PCL Hy_FN some depositions on the top of the fibers due to the presence of fibronectin. Similar roughness on the fibers surface was already reported in literature (Chutipakdeevong et al., 2014) and related to the functionalization with fibronectin. For PCL EN_FN it was not possible to detect similar morphology on the top of the fibers, but some deposits are visible and they could be related to EDC-NHS treatment and the following fibronectin functionalization. The coaxial sample also showed fibrillary morphology but the average fiber diameter is not homogeneous as for the neat PCL mats and on the top of the fibers is not possible to detect any deposit which could be comparable with the sample PCL Hy_FN.

**Figure 4:**
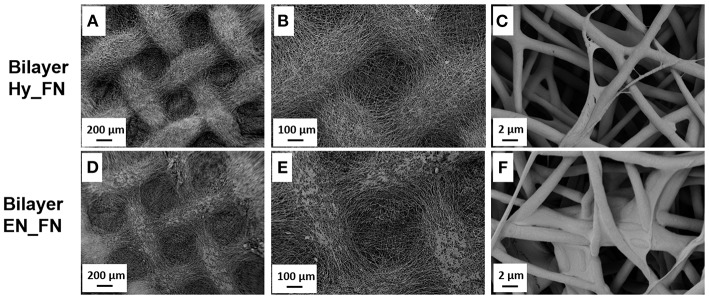
SEM micrographs with different magnification of bilayered scaffolds with the top layer functionalized with fibronectin by using the hydrolysis and protonation (Bilayer Hy_FN) **(A–C)** and EDC-NHS treatment (Bilayer EN_FN) **(D–F)**.

In Figure 4, it is reported the morphology of the bilayered scaffolds in which the first patterned layer was functionalized with fibronectin just after hydrolysis and protonation (Bilayer Hy_FN) and the other sample in which the first layer was functionalized after the treatment with EDC-NHS (Bilayer EN_FN). On Bilayer Hy_FN it is possible to observe similar deposits to sample PCL Hy_FN, while for Bilayer EN_FN similar deposits which seem to induce fiber merging are reported.

The morphological assessment confirmed that the fibrillary morphology of the PCL mats was preserved after hydrolysis, EDC-NHS treatment and fibronectin functionalization, regardless to the presence of pattern. The presence of fibronectin could be detected by the presence of deposits on the top of the fibers, while the EDC-NHS induced the precipitation of deposits difficult to remove by simply rinsing. In this case the patterned sample, because of its geometry, facilitates the rinsing process resulting free from these deposits (visible on the similar sample without pattern).

### ATR-FTIR Analysis

**Figure 5:**
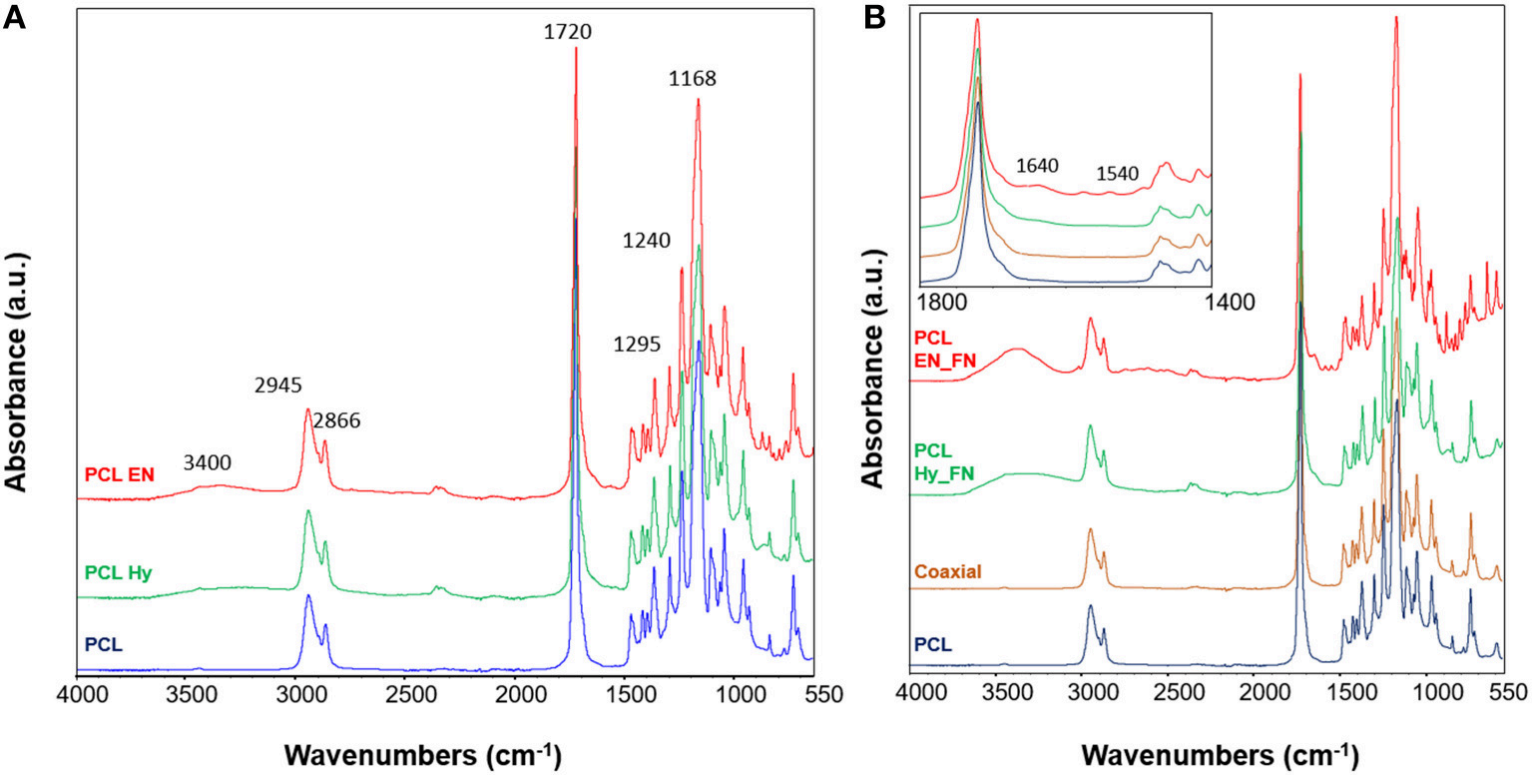
ATR-FTIR spectra of the scaffolds after hydrolysis and EDC_NHS treatment **(A)**; spectra of samples after the functionalization with fibronectin with three methods **(B)** with inset with a focus in the wavenumber range 1,800–1,400 cm^−1^. ATR-FTIR spectrum of neat PCL was used as control for all samples. Main bands discussed in the text are reported on the spectra.

ATR-FTIR analysis was performed to assess the effects of hydrolysis, EDC-NHS treatment and the presence of fibronectin after the functionalization of the scaffolds. In Figure 5A the spectra of PCL Hy and PCL EN are reported. The spectrum of neat PCL mats is included as control for the evaluation of hydrolysis and EDC-NHS treatment. It is possible to notice that all main bands ascribable to PCL structure are present in all spectra. In fact, it is possible to detect the peaks related to CH_2_ stretching centered at 2,945 and 2,866 cm^−1^, the stretching of carbonyl groups at 1,720 cm^−1^, C-O stretching at 1,295 cm^−1^ and C-O-C stretching at 1,240 and 1,168 cm^−1^ (Catledge et al., 2007; Ghasemi-Mobarakeh et al., 2008; Gaharwar et al., 2014). Moreover, due to the exposure of OH groups, it is possible to detect a broad band ascribable to OH vibrations centered at 3,400 cm^−1^.

Functionalized samples and coaxial scaffolds were also assessed and their spectra are reported in Figure 5B. Also in this case, for all samples the main bands ascribable to PCL (reported as control in the same plot) are prevalent with respect to the other contributions. In the inset a zoom view of the spectra in the range between 1,800 and 1,400 cm^−1^ is reported. It is possible to notice that the spectrum of the coaxial sample does not show significant differences or shifts with respect to the neat PCL, this result could be explain with the fact that the amount of fibronectin on the fiber surface is not enough to be well-detected with this technique and further characterization is needed. Also, for the other two methods, just for the sample PCL EN_FN it is possible to notice the peaks centered at 1,640 and 1,540 cm^−1^ ascribable to amide I and amide II, respectively (Chutipakdeevong et al., 2014). For the PCL EN_Hy it is just possible to detect some changes in the subtraction spectrum and by applying the deconvolution on the carbonyl peak (centered at 1,720 cm^−1^) (data not shown).

### Surface Analysis

**Figure 6:**
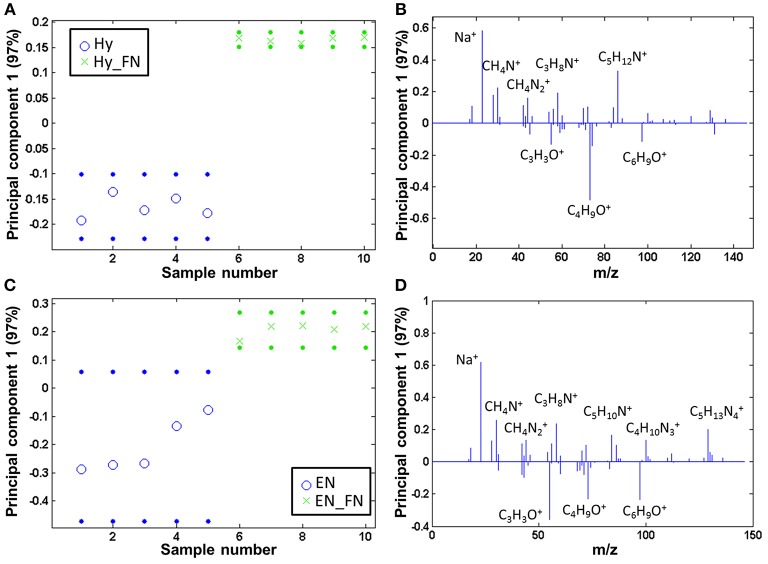
PCA comparing the fibronectin coated samples to linker coated PCL: scores (with 95% confidence limits; dotted lines) of **(A)** PCL Hy and **(B)** PCL EN mediated protein adsorption; **(C,D)** corresponding loadings.

Both PCL Hy and PCL EN modified fibers were coated with fibronectin and subsequently, the protein free and protein coated samples were analyzed by ToF-SIMS and compared by PCA. The samples are clearly separated and fragments related to amino acids are solely detectable on the fibronectin modified samples, both for PCL Hy_FN (scores are displayed in Figure 6A and loadings in Figure 6B) and PCL EN_FN (scores are displayed in Figure 6C and loadings in Figure 6D) mediated protein adsorption.

When comparing the ratio of the sums of the amino acid (*m/z* 30.04, amino acids, CH_4_N^+^; *m/z* 44.05, Ala/Asn/Leu, CH_4_${\text{N}}_{2}^{+}$; *m/z* 58.07, Glu, C_3_H_8_N^+^; *m/z* 84.08, Lys/Leu, C_5_H_10_N^+^; *m/z* 86.10, Ile/Leu, C_5_H_12_N^+^; *m/z* 100.09, Arg, C_4_H_10_${\text{N}}_{3}^{+}$; *m/z* 129.11, Arg, C_5_H_13_${\text{N}}_{4}^{+}$) and PCL characteristic fragments with the highest scores (*m/z* 55.02, C_3_H_3_O^+^; *m/z* 73.06, C_4_H_9_O^+^; *m/z* 97.07, C_6_H_9_O^+^), the PCL EN_FN samples reach a higher value than the PCL Hy_FN samples, namely 14.83 (± 3.35) vs. 1.92 (± 0.22). This indicates that the fibronectin coverage is higher if fibronectin is adsorbed to PCL via an EN linker.

**Figure 7:**
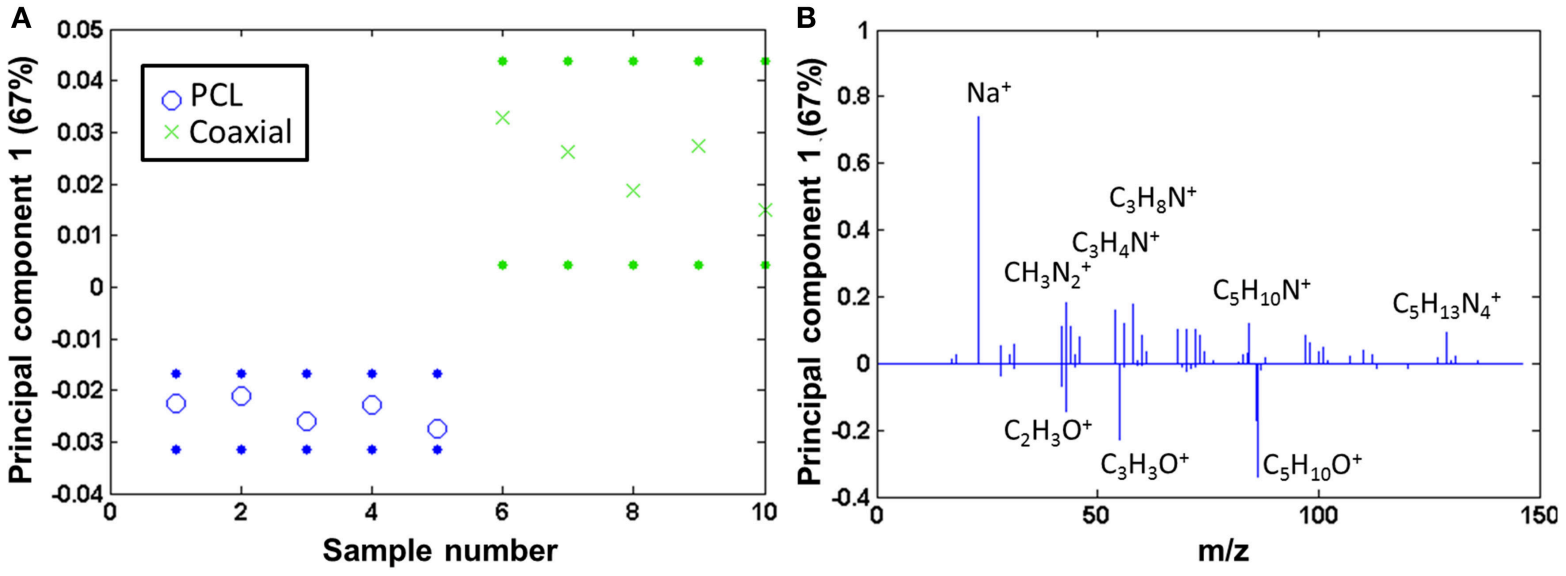
PCA comparing the coaxial samples to bare PCL: **(A)** scores (with 95% confidence limits; dotted lines) and **(B)** corresponding loadings.

The coaxial samples only seem to be covered by a comparatively low amount of fibronectin [amino acid to PCL ratio is 0.05 (± 0.01)]. The coaxial samples consequently were compared to pure PCL by PCA, and also here, fibronectin was clearly detectable on the coated fibers, whereas the neat PCL samples show mainly fragments of the type C_x_H_y_${\text{O}}_{\text{z}}^{+}$ (scores Figure 7A and loadings Figure 7B).

**Figure 8:**
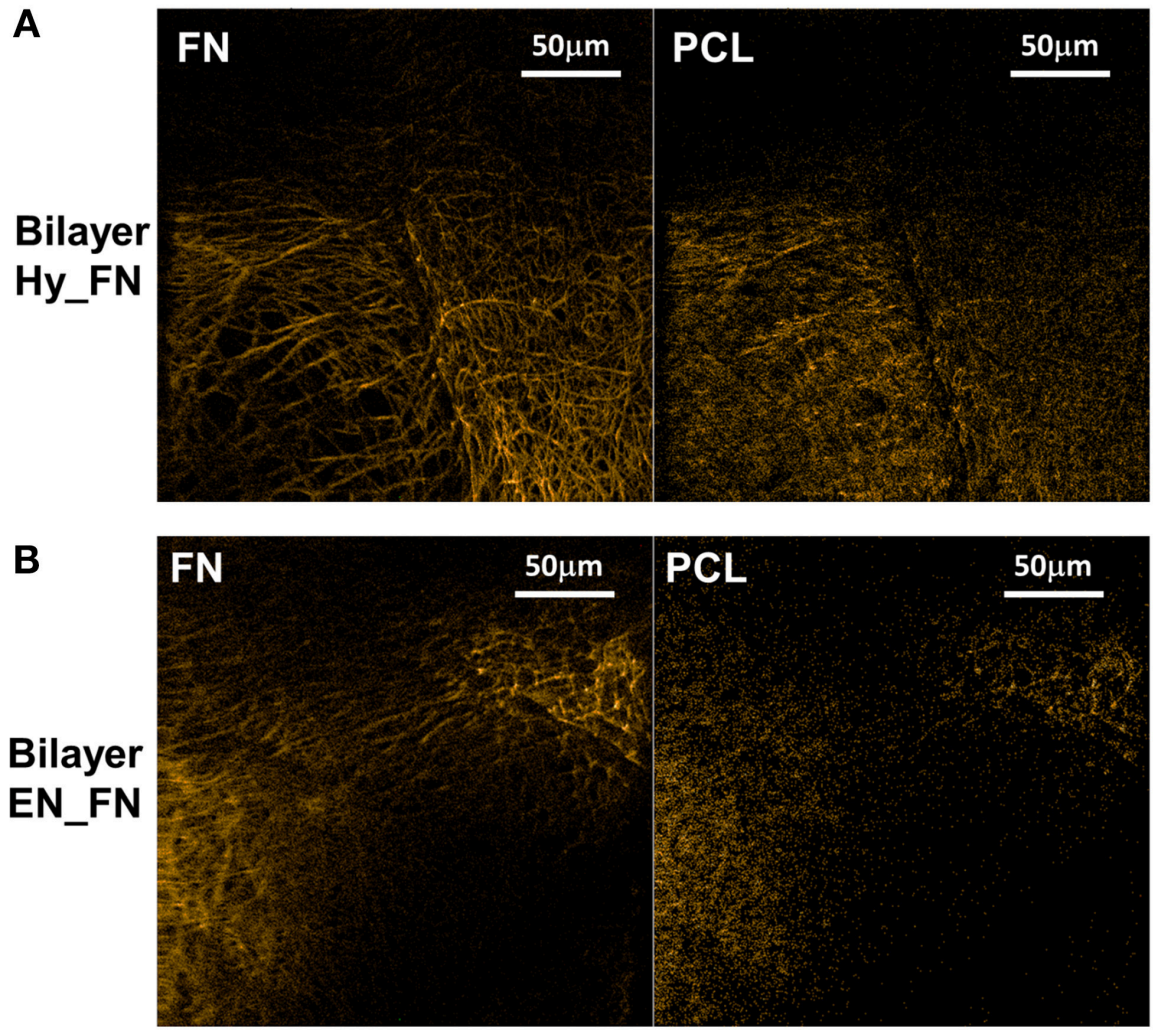
ToF-SIMS negative polarity images of the fragments characteristic for fibronectin (left) and PCL (right) for bilayer samples Hy_FN **(A)** and EN_FN **(B)**.

The bilayered samples were analyzed in ToF-SIMS by imaging, a corner of the top mesh was imaged for each sample. Negative polarity images were chosen for display, as they are less sensitive to topographical features. By PCA comparing neat PCL to the protein covered samples, CN^−^ (*m/z* 26.01) and CNO^−^ (*m/z* 42.00) were determined as related to fibronectin, whereas ${\text{CHO}}_{2}^{-}$ (*m/z* 45.00), C_2_H_2_${\text{O}}_{2}^{-}$ (*m/z* 58.01), C_3_H_3_${\text{O}}_{2}^{-}$ (*m/z* 71.03), and C_6_H_11_${\text{O}}_{3}^{-}$ (*m/z* 131.10) were assigned as characteristic fragments for bare PCL (cf. Supplementary Figure 1). Figure 8A shows overlays of the respective fragments for Bilayer Hy_FN samples (left, fibronectin; right, PCL characteristic fragments). Figure 8B shows the results for the Bilayer EN_FN samples accordingly.

Topography influences could not be completely removed in ToF-SIMS image evaluation. However, the protein related fragments clearly dominate on the top mesh in both cases, while the PCL related fragments occur more homogeneously over the entire structure.

### Fibronectin Release

**Figure 9:**
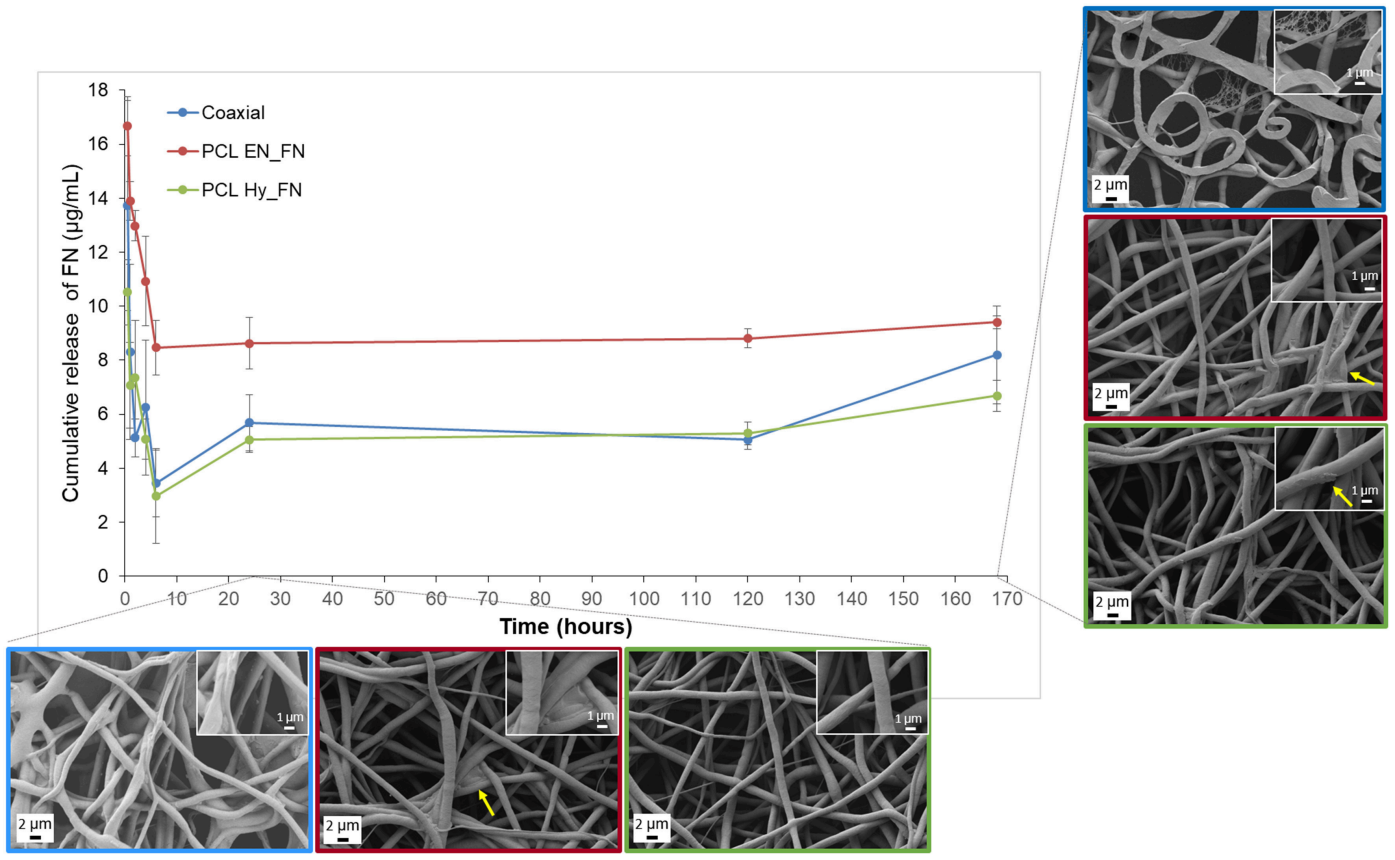
Fibronectin release profile and SEM micrographs of coaxial, PCL EN_FN and PCL Hy_FN samples after immersion in PBS.

The results obtained from the fibronectin release highlighted that the higher amount of protein was release in the first 6 h of immersion in PBS for all the samples, but the release is sustained till 7 days after immersion, confirming that the trend of the release is not dependent on the functionalization method, as reported in Figure 9. Among the three methods, it is possible to confirm that PCL EN_FN samples adsorbed (and therefore release) higher amount of protein through all the time point, as already showed with ToF-SIMS analysis. PCL Hy_FN and coaxial samples showed comparable values of release for all the time point. In Figure 9, besides the FN release, also a morphological investigation was performed for all samples after 1 and 7 days of immersion in PBS. The fibrillary morphology was not altered for all samples in both the analyzed timepoints. The flattened fibers reported in the coaxial sample after 7 days of immersion are related only to the investigated side of the sample, in fact the flattened fibers were the layer in direct contact with the collector and this shape is not related to the protein release. Nevertheless, in this sample, it is possible to observe a small net-in-the-net morphology, more clearly visible in the inset at higher magnification, ascribable to the release of protein from the polymeric fibers. For PCL EN_FN and PCL Hy_FN the presence of protein can still be detected after seven days of immersion on the fibers, as highlighted with yellow arrows on the SEM micrographs. In the existing literature on electrospun fibers functionalized with fibronectin, several approaches have been proposed for the evaluation and quantitation of the protein. In fact, some of these studies performed adsorption assays to confirm the presence of the protein on the mats by using modified enzyme linked immunosorbent assay (ELISA) (Mukhatyar et al., 2011) or by using human plasma fibronectin fluorescently labeled with DyLight 550 and followed by the analysis of the samples with fluorescence plate reader (Regis et al., 2014), while in this paper a novel approach of using ToF-SIMS analysis to confirm the presence of fibronectin on the mats and to compare the three different method was proposed. For the evaluation of the protein release and degradation, most of the studies used weight loss measurements (Campos et al., 2014; Chutipakdeevong et al., 2014), but for the samples fabricated in this study the differences between the measured weight of the samples before and after the functionalization were in the same range of the intrasample variability and close to the resolution value of the analytical balance. For this reason the weight loss was not considered to be an effective parameter to evaluate protein release and fiber degradation. For what concern the SEM analysis of functionalized samples after degradation also Campos et al. (2014) showed SEM micrographs of poly(d,l-lactide-co-glycolide) (PLGA) after immersion in PBS, showing morphological modifications which were not detected in our samples (as showed in Figure 9), also in the case of PCL Hy_FN which was obtained by using a similar protocol of functionalization.

### Cell Viability and Proliferation

**Figure 10:**
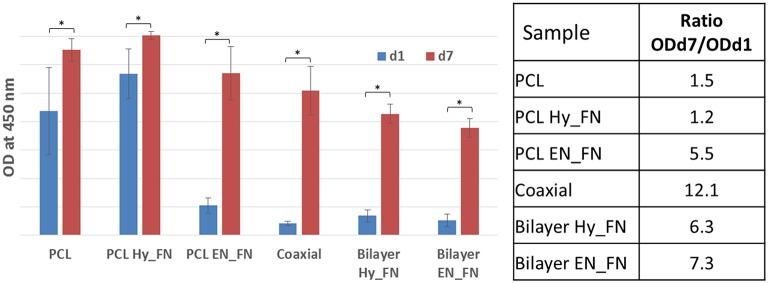
WST-8 analysis: histograms of OD at 450 nm for all samples 1 day and 7 days after the seeding. A table reporting the increase of the OD values expressed as ratio between the measured OD 7 days after the seeding respect to the OD measured after 1 day. Asterisk denotes significant difference, *p* < 0.05.

The stromal cell line ST-2, derived from mouse bone marrow was selected because it showed osteoblast (Otsuka et al., 1999; Koike et al., 2005), chondrocyte (Robins et al., 2005), and adipocyte differentiation (Ding et al., 2003; Huang et al., 2010), confirming to be a suitable cell line for preliminary assessment (cell viability and proliferation) for both soft and hard tissue engineering applications. The results obtained from cell biology studies highlighted important issues. WST-8 results reported in Figure 10 show that after 1 day from seeding, most of the functionalized samples, including the bilayered scaffolds, did not show significant higher values than the neat untreated PCL used as control. The only sample which showed comparable values of OD 1 day after the seeding is PCL Hy_FN. This result could be explained by the presence of residuals inside the mats after EDC-NHS treatment difficult to remove because of the dense fibrillary morphology of the samples, demonstrating better performance of the first method of functionalization (PCL Hy_FN sample), with significant higher OD values respect to the other functionalized samples after 1 day of cell culture. The coaxial sample did not show significant higher results in term of cell adhesion 1 day after seeding, with respect to untreated PCL, most probably because of the low amount of fibronectin on the fibers surface and the higher distribution in the average fiber diameter.

Another interesting result is represented by the increase in the OD values measured seven days after the seeding. To highlight this result, a table with the ratio between OD measured after seven days and OD measured 1 day after the seeding is reported in Figure 10. These ratios highlighted that in all samples an increase in OD values was measured, which correlates well with the cell proliferation on all scaffolds. All functionalized samples, beside PCL Hy_FN, showed higher values with respect to the control. The highest value was calculated for the coaxial sample, showing that even if the amount of fibronectin could be not sufficient to have a significant improvement in cell adhesion, this does not inhibit the proliferation capability of the cells. Moreover, an important role is also played by the morphology and the inhomogeneity of the fibrillary structure.

For what concerns the bilayered scaffolds, it was not possible to detect significant difference in their behavior neither in terms of cell adhesion, nor for cell proliferation. The ratio between the measured OD values is also comparable and higher with respect to the single layered scaffolds treated with the same functionalization methods. This result is pivotal to understand the relevance of the graded scaffolds in terms of morphology and functionalization for supporting cell proliferation.

**Figure 11:**
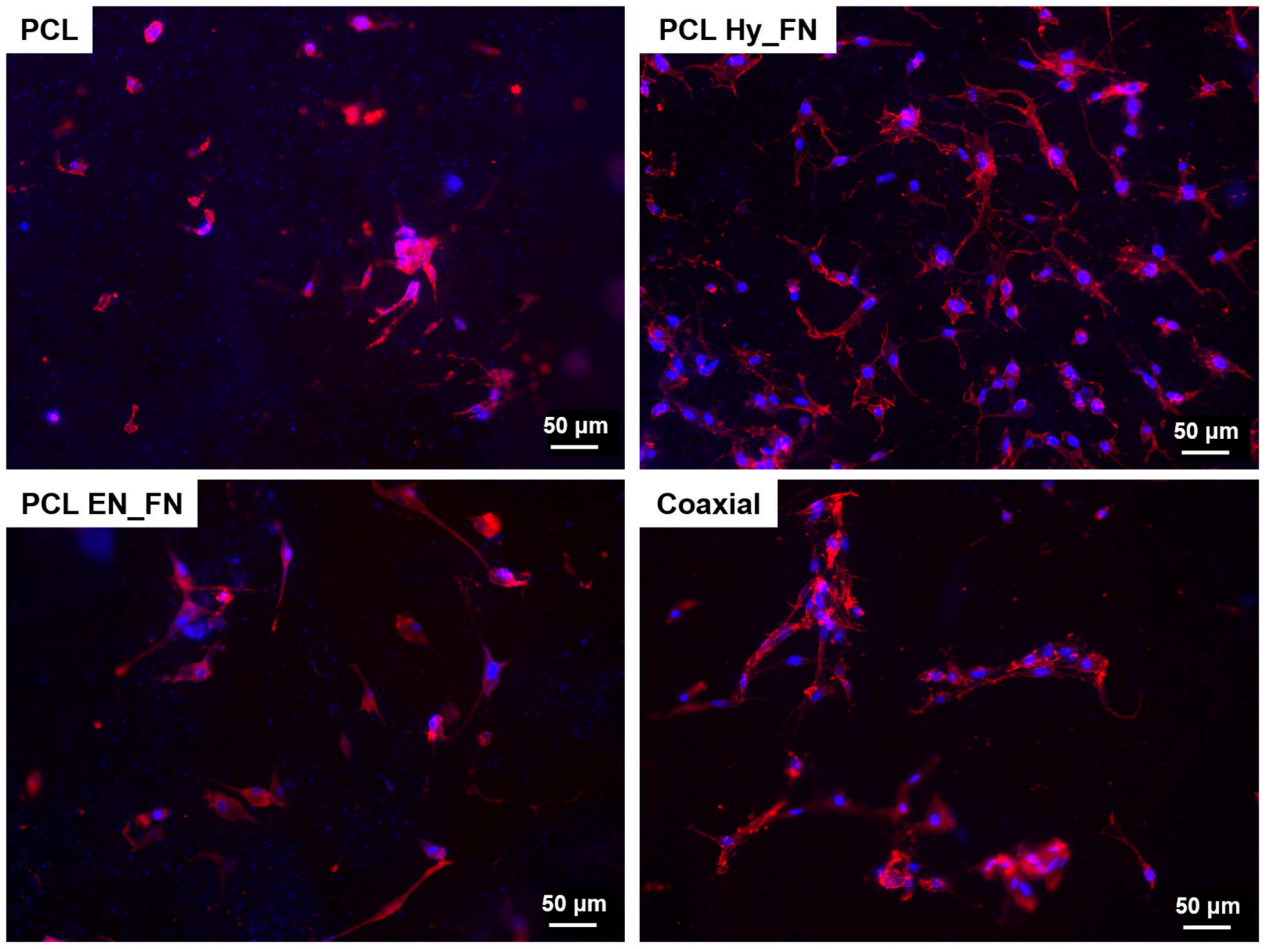
Fluorescence images of actin filaments (red) and cell nuclei (blue) for the samples PCL, PCL Hy_FN, PCL EN_FN, and coaxial 7 days after the seeding.

Fluorescence staining performed seven days after seeding confirmed the results obtained by WST-8 test. In particular, it is possible to detect cells attached on the top of all electrospun mats, as reported in Figure 11. Moreover, despite results showing that samples functionalized with EDC-NHS chemistry allowed the immobilization of a higher amount of protein, PCL Hy_FN showed the highest density of nuclei and actin filaments oriented through the fibers direction. These results could be explained with the presence of some residuals of the functionalization process which could justify the differences detected in cell morphology.

**Figure 12:**
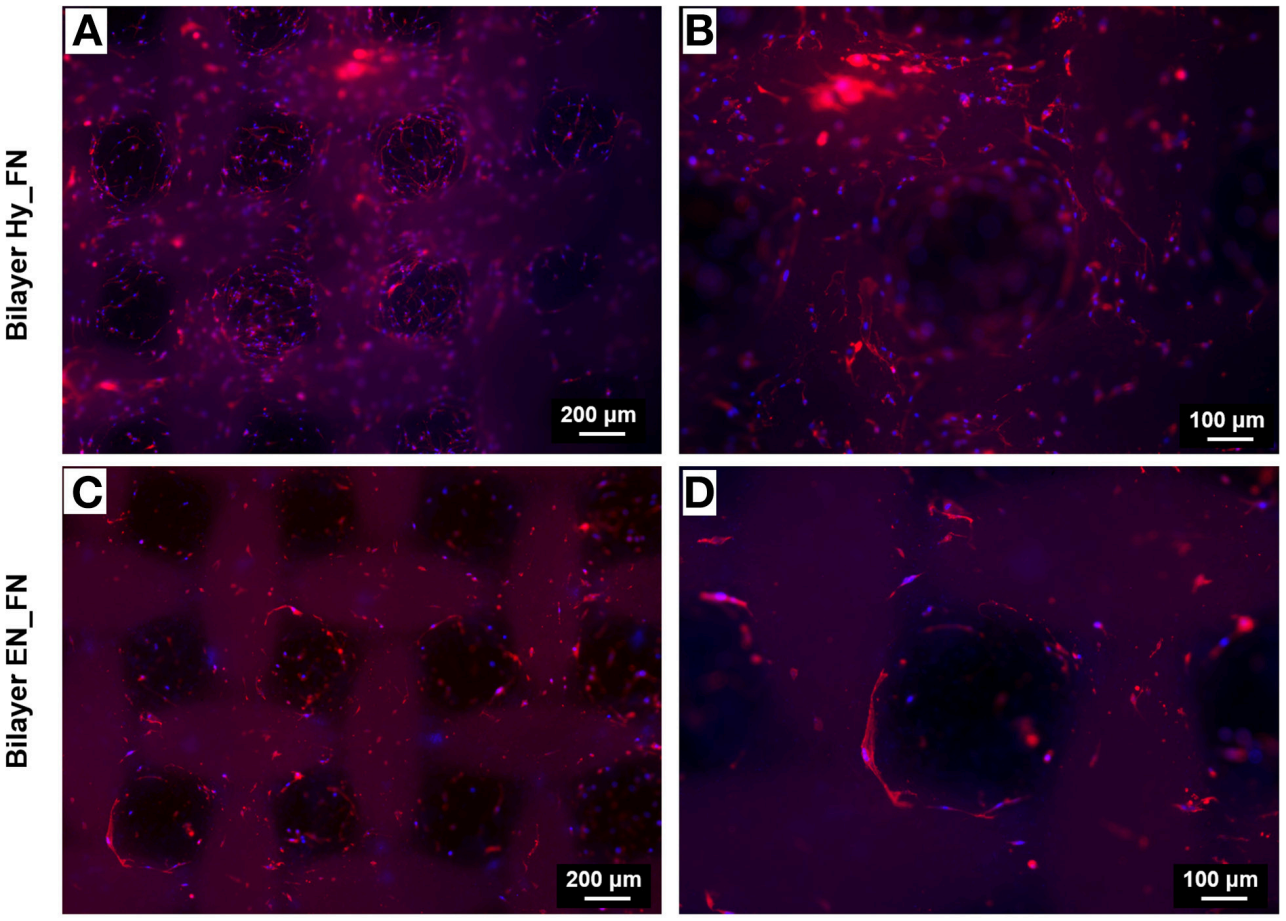
Fluorescence images of actin filaments (red) and cell nuclei (blue) for the bilayered samples Bilayer Hy_FN **(A,B)** and Bilayer EN_FN **(C,D)** with two magnifications.

For the bilayered scaffolds, the results related to the staining of nuclei and actin filaments are shown in Figure 12. In particular, two different magnifications are reported to highlight how the presence of a patterned top layer affects the direction of cell adhesion and proliferation. From the results in Figure 12, making a comparison between the two functionalization techniques, Bilayer Hy_FN looks characterized by higher density of cells, visible by the nuclei stained in blue. In both samples, it is possible to confirm that cells proliferated following the pore geometry and the pattern improved also the infiltration of cells through the pores, as it is possible to notice in Figures 12B,D. The cell alignment following the pattern morphology could be enhanced by the presence of fibronectin in the top layer, since in literature the role of fibronectin as topographical signal on electrospun fibers is already reported (Mukhatyar et al., 2011). These results are relevant, since an optimal cell infiltration in electrospun scaffolds is complex to achieve and it still represents a challenge faced with several techniques (Rnjak-Kovacina and Weiss, 2011).

## Conclusions

In the present work PCL electrospun mats were successfully functionalized with fibronectin by using three different functionalization methods. A comparative analysis was performed in terms of efficacy of functionalization processes, influence on fiber morphology and cell response. Results showed satisfactory results for all the functionalized samples, with respect to the untreated PCL, but better performance in terms of cell response were achieved by the sample functionalized with the surface entrapment (after hydrolysis), which results to be the “best way” to achieve effective functionalization for electrospun mats. Further optimizations are still needed for the sample fabricated with the coaxial electrospinning, because the investigated conditions (kept constant for the assessment of the comparative study) are not the optimal conditions with sufficient amount of protein on fibers surface as well as the inhomogeneity in fiber diameter distribution affected the detection of protein with ATR- FTIR analysis and cell response. The most promising results were obtained with the bilayered scaffolds. In fact, the presence of a pattern improved cell infiltration through the electrospun scaffold and it also confirmed that the functionalization achieved with surface entrapment method was more effective than the other. The obtained results are particularly relevant for all of the applications of electrospun mats for tissue engineering purpose, either for soft tissue or interface tissues.

## Data Availability

The raw data supporting the conclusions of this manuscript will be made available by the authors, without undue reservation, to any qualified researcher.

## Author Contributions

LL designed the experiments, performed the experimental activities related to the electrospun fibers fabrication, functionalization, cell tests, and she wrote the first draft of the manuscript. MK performed the ToF-SIMS characterization, data analysis, and contributed in writing the manuscript. AB advised during the project progress, discussed the results, and collaborated in writing the manuscript. All the authors read and approved the manuscript.

### Conflict of Interest Statement

The authors declare that the research was conducted in the absence of any commercial or financial relationships that could be construed as a potential conflict of interest.
