# Supplementary material for: Fibronectin Functionalized Electrospun Fibers by Using Benign Solvents: Best Way to Achieve Effective Functionalization
Source: Front Bioeng Biotechnol. 2019 Apr 3;7:68. doi: 10.3389/fbioe.2019.00068 (PMC6456675; doi:10.3389/fbioe.2019.00068)
Supplement: Supplementary file 1 [file Data_Sheet_1.PDF]

Supplementary Material

1 Peak lists for positive polarity spectra

A peak list containing amino acid (AA) and linker fragments was compiled according to reference (Killian, Manuela S., Adam J. Taylor, and David G. Castner. 2018. “Stabilization of Dry Protein Coatings with Compatible Solutes.” Biointerphases 13 (6): 06E401) and is shown in Supplementary Table 1. Fragments that were too close to produce clearly distinguishable signals were combined. PCA and the linkers produce fragments that overlap with some of the amino acid signals. These signals were assigned by comparing linker coated PCL to samples with fibronectin via PCA. All signals loading with the linker samples in the respective first principle component (which separates the two sample types) are marked with an “x” in Table S1.

Supplementary Table 1: Peak lists used for PCA analysis of positive polarity spectra. Signals loading with the linker coated PCL are marked with an “x”.

Mass	Ion	Assignment to specific AAs	PCL Hy_FN	PCL EN_FN
17.0269	NH_3^+	amino acids		
18.0362	NH_4^+	amino acids		
22.9922	Na^+	salt		
28.0197	CH_2N^+	Leu + others		
30.0386	CH_4N^+	Leu, Gly + others		
31.0185	CH_3O^+	PCL	x	x
31.0449	CH_5N^+	amino acids		
42.0098	$C_2H_2O^+$	PCL	x	x
42.0393	$C_2H_4N^+$	Ala, Gly , His, Leu, Ser		
43.0178	$C_2H_3O^+$	PCL	x	x
43.0287	$CH_3N_2^+$	Arg		
43.0405	$C_2H_5N^+$	Ala, Leu, Ser		
44.0375	$CH_4N_2^+$	Arg		
44.0527	$C_2H_6N^+$	Ala, Asn, Leu		
44.9784	CHS^+/CHO_2^+	Cys/PCL	x	x
44.9963	CHO_2^+	PCL	x	x

45.9908	CH_2S^+	Cys		
54.0409	$C_3H_4N^+$	His		
55.0211	$C_3H_3O^+$	PCL	x	x
56.0224	$C_3H_4O^+$	PCL	x	x
56.0497	$C_3H_6N^+$	Lys, Met, Val		
58.0328	$C_2H_4NO^+/C_3H_6O^+$	Ser, Gly/PCL	x	
58.0682	$C_3H_8N^+$	Glu		
58.9972	$C_2H_3S^+/C_2H_3O_2^+$	Cys/PCl	x	
59.0513	$CH_5N_3^+/C_3H_7O^+$	Arg/PCL	x	x
60.0216	$C_2H_4O_2^+$	PCL	x	x
60.0477	$C_2H_6NO^+$	Ser		
61.0124	$C_2H_5S^+$	Met		
68.0584	$C_4H_6N^+/C_5H_8^+$	Pro (, Lys)/PCL	x	x
69.0365	$C_4H_5O^+$	Thr/PCL	x	x
70.0303	$C_3H_4NO^+/C_4H_6O^+$	Asn/PCL	x	x
70.0727	$C_4H_8N^+$	Pro, Val, Arg, Leu		
71.0119	$C_3H_3O_2^+$	Ser/PCL	x	x
72.0434	$C_3H_6NO^+$	Gly		
72.0825	$C_4H_{10}N^+$	Val		
73.0637	$C_2H_7N_3^+/C_4H_9O^+$	Arg/PCL	x	x
74.0646	$C_3H_8NO^+/C_4H_{10}O^+$	Thr/PCL	x	x
76.0240	$C_2H_6SN^+/C_3H_8O_2^+$	Cys/PCL	x	x
82.0521	$C_4H_6N_2^+$	His		
83.0740	$C_5H_7O^+$	Val/PCL	x	x
84.0432	$C_4H_6NO^+$	Gln, Glu		
84.0825	$C_5H_{10}N^+$	Lys, Leu		
86.0990	$C_5H_{12}N^+$	Ile, Leu		
87.0562	$C_3H_7N_2O^+$	Asn		
88.0401	$C_3H_6NO_2^+$	Asn, Asp		
97.0717	$C_6H_9O^+$	PCL	x	x
98.0589	$C_4H_4NO_2^+$	Asn		
100.0900	$C_4H_{10}N_3^+$	Arg		

101.0956	$C_4H_{11}N_3^+$	Arg		
102.0505	$C_4H_8NO_2^+$	Glu		
107.0468	$C_7H_7O^+$	Tyr		
110.0863	$C_5H_8N_3^+$	His, Arg		
112.0928	$C_5H_{10}N_3^+$	Arg		
113.0184	$C_4H_3NO_3^+$	NHS		x
120.0800	$C_8H_{10}N^+$	Phe		
127.0972	$C_5H_{11}N_4^+$	Arg		
129.1140	$C_5H_{13}N_4^+$	Arg		
130.0608	$C_9H_8N^+$	Trp		
131.0627	$C_9H_7O^+$	Phe/PCL	x	
136.0784	$C_8H_{10}NO^+$	Tyr		

2 PCA of negative polarity spectra

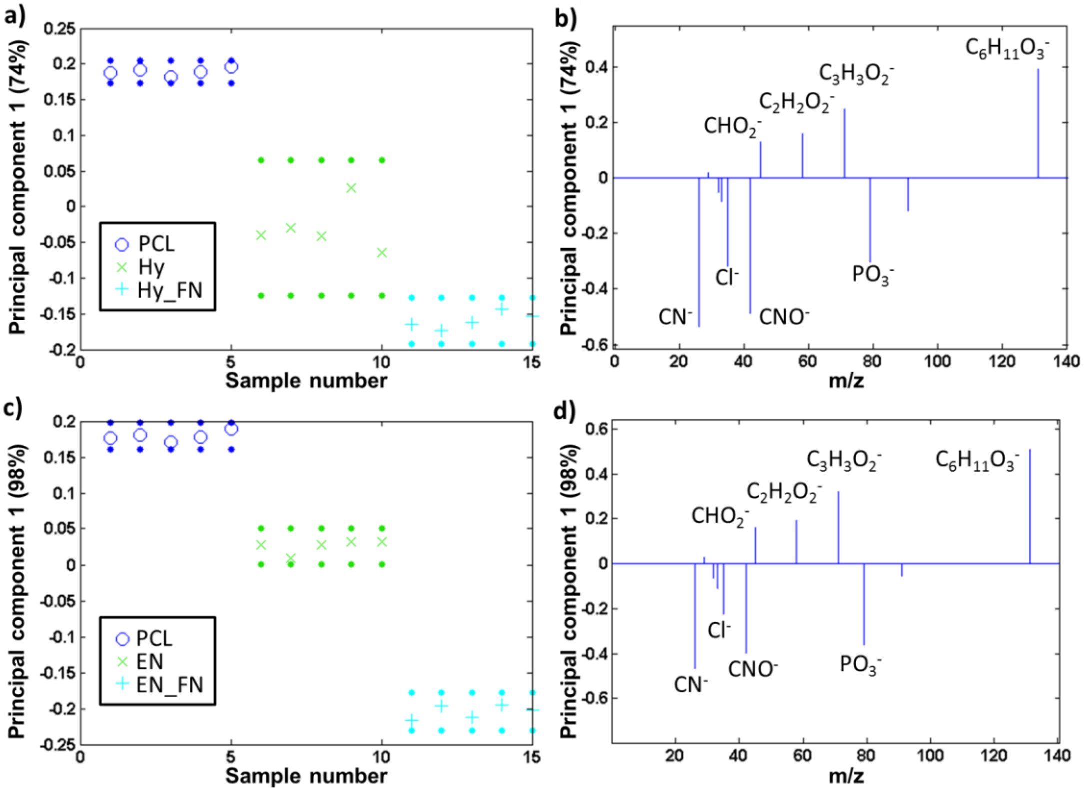

Supplementary Figure 1: Scores (with 95% confidence limit; dotted lines) and loadings of negative polarity spectra. a) and b) PCA separating PCL, PCL Hy and PCL Hy_FN samples; c) and d) PCA separating PCL, PCL EN and PCL EN_FN samples.
